# QTL mapping and BSA-seq map a major QTL for the node of the first fruiting branch in cotton

**DOI:** 10.3389/fpls.2023.1113059

**Published:** 2023-01-25

**Authors:** Xiaoyun Jia, Shijie Wang, Hongxia Zhao, Jijie Zhu, Miao Li, Guoyin Wang

**Affiliations:** Institution of Cereal and Oil Crops, Hebei Academy of Agriculture and Forestry Sciences/Hebei Laboratory of Crop Genetics and Breeding/Hebei Key Laboratory of Crop Cultivation Physiology and Green Production, Shijiazhuang, China

**Keywords:** cotton earliness, node of the first fruiting branch, QTL mapping, BSA-seq, candidate gene

## Abstract

Understanding the genetic basis of the node of the first fruiting branch (NFFB) improves early-maturity cotton breeding. Here we report QTL mapping on 200 F_2_ plants and derivative F_2:3_ and F_2:4_ populations by genotyping by sequencing (GBS). BC_1_F_2_ population was constructed by backcrossing one F_2:4_ line with the maternal parent JF914 and used for BSA-seq for further QTL mapping. A total of 1,305,642 SNPs were developed between the parents by GBS, and 2,907,790 SNPs were detected by BSA-seq. A high-density genetic map was constructed containing 11,488 SNPs and spanning 4,202.12 cM in length. A total of 13 QTL were mapped in the 3 tested populations. JF914 conferred favorable alleles for 11 QTL, and JF173 conferred favorable alleles for the other 2 QTL. Two stable QTL were repeatedly mapped in F_2:3_ and F_2:4,_ including *qNFFB-D3-1* and *qNFFB-D6-1*. Only *qNFFB-D3-1* contributed more than 10% of the phenotypic variation. This QTL covered about 24.7 Mb (17,130,008–41,839,226 bp) on chromosome D3. Two regions on D3 (41,779,195–41,836,120 bp, 41,836,768–41,872,287 bp) were found by BSA-seq and covered about 92.4 Kb. This 92.4 Kb region overlapped with the stable QTL *qNFFB-D3-1* and contained 8 annotated genes. By qRT-PCR, *Ghir_D03G012430* showed a lower expression level from the 1- to 2-leaf stage and a higher expression level from the 3- to 6-leaf stage in the buds of JF173 than that of JF914. *Ghir_D03G012390* reached the highest level at the 3- and 5-leaf stages in the buds of JF173 and JF914, respectively. As JF173 has lower NFFB and more early maturity than JF914, these two genes might be important in cell division and differentiation during NFFB formation in the seedling stage. The results of this study will facilitate a better understanding of the genetic basis of NFFB and benefit cotton molecular breeding for improving earliness traits.

## Introduction

Upland cotton (*Gossypium hirsutum* L. AADD, 2n=52) is the most important fiber crop in the world, accounting for more than 90% of global cotton production (Chen et al., 2007; Ma et al., 2021). Cottonseed is also a good source of edible oil and vegetable protein (Zhang et al., 2015). Thus, upland cotton has significant value in dealing with the increasing human population. Earliness is one of the vital breeding goals to meet the needs of mechanism practice, especially during cotton harvesting (Jia et al., 2016; Li et al., 2021). Besides, early-maturity cotton, also known as short-season cotton, has many advantages in inter-cropping between cereal crops and cotton to increase land utilization efficiency in China (Cheng et al., 2021; Zhao et al., 2022). Earliness is a typical characteristic of early-maturity cotton. As yield and fiber quality have dominated cotton breeding for decades, little attention has been paid to earliness.

In terms of plant development, cotton earliness is described as flowering time (FT), whole growth period (WGP), and flowering-to-boll opening period (FBP) (Richmond and Radwan, 1962; Li et al., 2020). Plant height (PH), node of the first fruiting branch (NFFB), and height of NFFB (HNFFB) are also important indexes for earliness (Godoy and Palomo, 1999; Jia et al., 2016). NFFB is the most reliable index in terms of indicating cotton earliness, which has better consistency among environments, and significantly positively correlates with FT, WGP, PH, and HNFFB (Guo et al., 2008; Su et al., 2016; Zhang et al., 2021). All six traits mentioned above have relatively high broad-sense heritabilities and significant environmental influences (Jia et al., 2016; Li et al., 2020; Li et al., 2021).

Several studies for cotton earliness genetic detection through QTL mapping and GWAS analysis have been published (Li et al., 2020). Guo et al. (2008); Guo et al., 2009 mapped QTL for NFFB in two F_2_ populations and used the results to measure flowering time. Li et al. (2013) mapped 54 QTL for cotton earliness in two F_2_ and their F_2:3_ populations, and a common QTL for the budding period could explain 12.6% of the phenotypic variation. Benefiting from high-throughput sequencing techniques and high-quality genome sequences of TM-1 and NDM8, the efficiency and accuracy of QTL mapping and GWAS analysis have been significantly improved (Li et al., 2015; Zhang et al., 2015; Hu et al., 2019; Wang et al., 2019; Ma et al., 2021). Jia et al. (2016) constructed a high-density genetic map containing 6295 SNP and 139 SSR markers for a RIL population by RAD-seq, mapped 247 QTL for cotton earliness in six consecutive years, and found an extremely prominent chromosome region on D3 with six stable major QTL. Li et al. (2017) constructed a SNP-based genetic map for an F_2_ population by GBS, mapped 47 QTL for cotton earliness, and found a major region on D3 overlapping with the results of Jia et al. (2016). Su et al. (2016) developed 81,675 SNPs in 355 upland cotton accessions; 13 significant associations between SNP and earliness traits were found by GWAS, a major locus and a candidate gene were also mapped on D3. Li et al. (2021) re-sequenced 436 cotton accessions and developed 10,118,884 SNPs and 864,132 InDels; 307 significant SNPs were found for cotton earliness by GWAS, including 43 SNPs in a 3.7 Mb region on D3 overlapping with previous results. The reports mentioned above imply the significant role of chromosome D3 in controlling cotton earliness, which has been emphasized again by Ma et al. (2018) and Zhang et al. (2021). Besides, Li et al. (2018) developed 49,650 SNPs in 169 upland cotton accessions by CottonSNP80K array; 29 significant SNPs and two candidate genes were found for cotton earliness. However, QTL fine mapping for cotton earliness, especially NFFB, has rarely been reported until now, and the genetic basis under earliness traits is still unclear.

This study used a nationally certified variety, Jifeng914 (JF914), with about 120 d WGP and 8 NFFB as the maternal parent, an early maturity inbred line Jifeng173 (JF173) with about 108 d WGP and 5 NFFB was used as the paternal parent. QTL mapping was conducted based on a high-density genetic map for an F_2_ population. The BC_1_F_2_ population was constructed and used for QTL mapping by BSA-seq. One stable QTL for NFFB spanning 24.7 Mb was shortened to 92.4 Kb. Eight genes were annotated in this core region and 2 genes with different expression patterns in the buds of JF173 and JF914 might be the candidates.

## Materials and methods

### Experimental materials and phenotypic trait

Jifeng 914 (JF914) (a larger phenotype cultivar with about 120 d WGP and 8 NFFB) was crossed with Jifeng 173 (JF173) (a smaller phenotype inbred line with about 108 WGP and 5 NFFB). An F_2_ population containing 417 plants was developed at Shijiazhuang in 2019; 200 plants from the F_2_ were randomly selected and continuously self-pollinated to F_2:3_ and F_2:4_ generations. The F_2:3_ and F_2:4_ populations were planted with two replicates in a completely randomized block design at Shijiazhuang in 2020 and 2021. One F_2:4_ line with low NFFB and a similar phenotype to JF914 was chosen and backcrossed with JF914 in 2021. And 23 BC_1_ plants were self-pollinated at Hainan in the winter of 2021 to construct the BC_1_F_2_ population containing 561 plants, which was planted in 2022 at Shijiazhuang. The materials were planted in single lines (5 m long and 70 cm between adjacent lines), and conventional field management was carried out.

The node of the first fruiting branch (NFFB) was tested. Every plant in the F_2_ and BC_1_F_2_ was measured. Ten plants in the middle of each line were measured in the F_2:3_ and F_2:4_ populations. Excel 2010 and SPSS 17 were used for data analysis.

### DNA sequencing

Genomic DNA was extracted by the CTAB method (Paterson et al., 1993). The genotyping-by-sequencing (GBS) method was applied for the F_2_ plants as detailed by Zhang et al. (2016); Li et al. (2017), and Zhou et al. (2016). Briefly, DNA was incubated at 37°C with *Mse* I (New England Biolabs, NEB), T4 DNA ligase (NEB), ATP 9NEB, and *Mse* Y adapter N containing barcodes. *Hae* III and *Rco*R I (NEB) were added into the *Mse*I digestions to further digest the fragments at 37°C. Fragments of 397–420 bp were purified and paired-end 150-bp sequenced on the Illumina HiSeq™ platform. High-quality reads were filtered based on (1) removing reads with ≥ 10% unidentified nucleotides (N); (2) removing reads with > 50% based on having Phred quality < 5; (3) removing reads with 10 nt aligned to the adapter, allowing ≤ 10% mismatches; and (4) removing reads containing *Hae* III or *Eco*R I.

For BSA-seq, 30 high NFFB plants and 30 low NFFB plants were selected from the BC_1_F_2_ population; the DNA of each plant was extracted and mixed to construct two DNA pools (high and low). Four samples were subjected to re-sequencing, including JF914, JF173, and high and low DNA pools. The GenoBaits DNA-seq Library Prep kit was used for library construction. First, 4 μl of GenoBaits End Repair Buffer and 2.7 μl of GenoBaits End Repair Enzyme were added to 200 ng DNA and incubated for 20min at 37°C and 20min at 72°C. Second, 2 μl of GenoBaits Ultra DNA ligase, 8 μl of GenoBaits Ultra DNA ligase Buffer, and 2 μl of GenoBaits Adapter were added and incubated for 30 min at 22°C. Third, fragments were purified by adding 48 μl of Beckman AMPure XP Beads. Fragments of 200–300 bp were reserved and sequenced on the MGI-2000/MGI-T7 planform. High-quality reads were filtered based on (1) removing the adaptor; (2) removing reads with >10% N; and (3) removing reads with >40% low-quality bases (Q ≤ 20).

The BWA software was used to align the clean reads against the reference genome of TM-1 (Wang et al., 2019). The GATK software was used for variation calling (Mckenna et al., 2010). SnpEff and ANNOVAR software were used for annotation (Wang et al., 2010; Cingolani et al., 2014).

### QTL mapping

Polymorphic markers developed from the F_2_ population were classified into eight segregation patterns (aa×bb, ab×cc, ab×cd, cc×ab, ef×eg, hk×hk, lm×ll, nn×np), and the aa×bb pattern SNPs were chosen to construct the genetic map. SNPs with segregation distortion (p<0.001) or integrity (<40%) or in the same reads or abnormal bases were filtered. SNP markers were sorted into 26 chromosomes according to their physical position on the reference genome. And then, the genetic map was constructed chromosome by chromosome using JoinMap 4.0 with a LOD score threshold of 6.0–20.0. The ICIM method in the QTL IciMapping software was used to detect QTL (Li et al., 2007). Parameters were set as 1 cM per step, PIN=0.001, and the LOD score was determined by a 1000 permutation test.

The Δ(SNP-index) and ED (Euclidean distance) methods were used to analyze the candidate region between the pools. The parameters of SNP-index and Δ(SNP-index) were calculated as follows: SNP-index(high) = Mhigh/(Mhigh + Phigh), SNP-index(low) = Mlow/(Mlow + Plow), and Δ(SNP-index) = SNP-index(low) – SNP-index(high). The M and P parameters represent the sequencing depth in JF914 and JF173, respectively. The parameters of ED were calculated as follows:

ED=


$$\sqrt{{(\text{Ahigh-Alow)}}^{2}+{\text{(Thigh-Tlow)}}^{2}+{\text{(Chigh-Clow)}}^{2}+{\text{(Ghigh-Glow)}}^{2}}$$

A, T, C, and G are the four base types. Ahigh, Thigh, Chigh, and Ghigh are the frequency of relevant bases in the high pool. Alow, Tlow, Clow, and Glow are the frequency of relevant bases in the low pool. The ED^4^ was used to eliminate background noise. The median+3SD was used as the threshold.

### qRT-PCR

For JF914 and JF173, total RNA was extracted from the bud and leaf at 1- to 6-leaf stages using a Plant RNA Purification Kit (Tiangen, Beijing, China). First-strand cDNA was reverse transcribed from 1 μg total RNA using a FastKing gDNA Dispelling RT SuperMix Kit (Tiangen, Beijing, China). qRT-PCR was carried out with the SYBR Premix Ex Taq (TAKARA, Japan) on a LightCycler480 instrument (Rotkreuz, Switzerland).

## Results

### Phenotypic variation

The NFFB of JF914 (7.8–8.5) is significantly bigger than that of JF173 (5.1–5.5). The maximum and minimum values of NFFB in the F_2_, F_2:3_, and F_2:4_ populations reveal transgressive segregation (**Table 1**). The mean value of NFFB in the segregation populations lies within the range of the two parents. Based on the absolute values of skewness and kurtosis, NFFB showed an approximately normal distribution.

**Table 1:** The statistics of NFFB in the parents, F_1_, and segregated populations.

Trait	JF914	JF173	F_2_					F_2:3_					F_2:4_				
			Max	Min	Mean	Skew	Kurt	Max	Min	Mean	Skew	Kurt	Max	Min	Mean	Skew	Kurt
NFFB (cm)	7.8-8.5^**^	5.1-5.5	9.0	5.0	6.6	-0.4	0.3	8.1	5.5	6.6	-0.2	0.3	8.7	5.1	6.3	0.1	0.3

NFFB, the node of the first fruiting branch; Max, maximum; Min, minimum; Skew, skewness; Kurt, kurtosis; ^**^, p<0.01.

### Sequence data and quality

A total of 416 G sequence data was obtained by genotyping by GBS, with an average of 25.91 G and 9× depth in the parents, 1.82 G and 0.7× depth in the F_2_ plants. The Q30 score reached 95.68%. And 99.63% of the F_2_ sequence data was successfully mapped to the reference genome, with an average coverage rate of 14.81% (Additional file 1).

A total of 318.81 G sequence data was obtained by re-sequencing for the four samples (**Table 2**). The sequence depths of the pools reached 31×, and Q30 scores are larger than 90%. More than 88% of the reference genome was covered.

**Table 2:** Sequence data of the parents and pools.

Sample	Raw Bases (bp)	Clean Bases (bp)	Q20 (%)	Q30 (%)	Align rate (%)	Average depth (×)	Coverage (%)
high	100,641,873,900	99,983,581,254	96.58	90.50	78.73	31.99	88.17
low	143,699,754,900	142,505,777,730	96.66	91.09	79.36	34.63	88.40
JF914	26,169,828,600	26,013,215,006	96.4	89.87	81.46	8.77	86.80
JF173	48,300,015,000	47,983,815,204	96.63	90.63	81.10	15.62	87.67

### Genetic map construction

**Figure 1:**
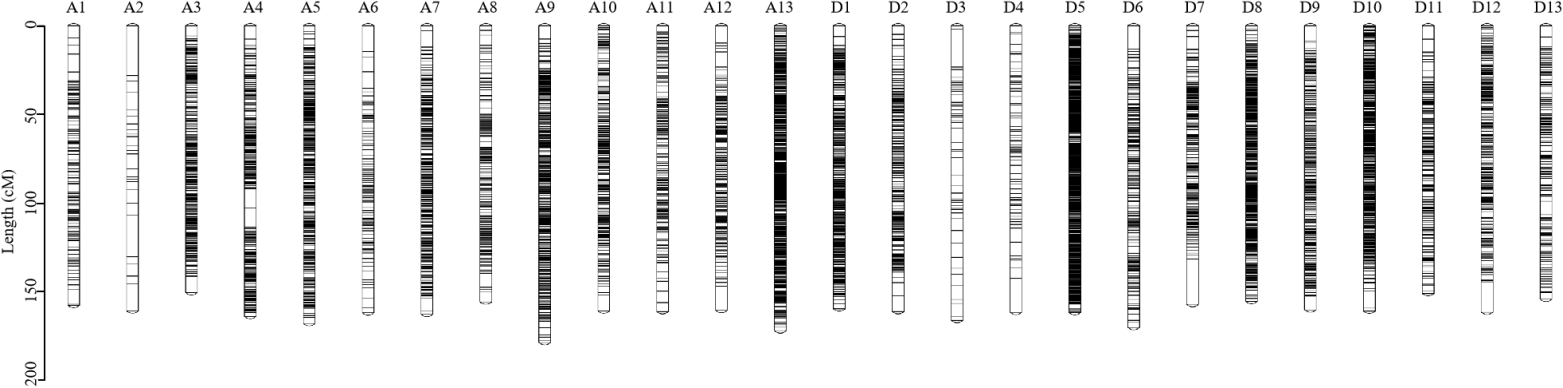
Marker distribution of the constructed genetic map.

**Figure 2:**
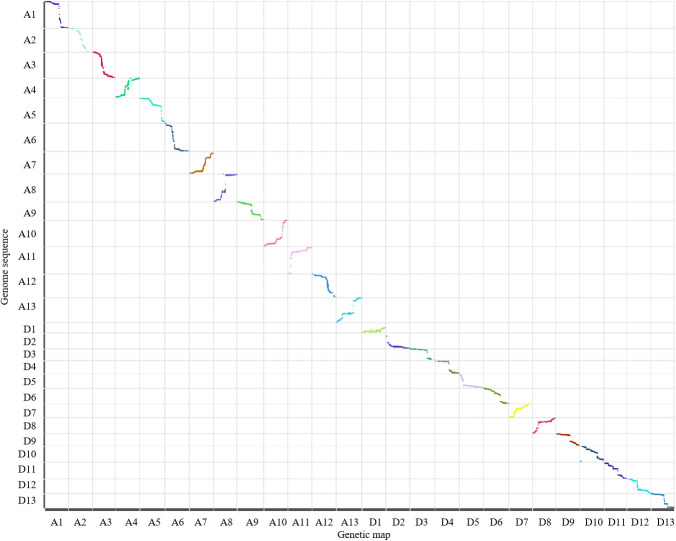
Colinearity analysis between the genetic map and reference genome sequence.

A total of 1,305,642 SNPs were developed between the parents, and 7 SNP types were detected (**Table 3**). Only the SNP in aa×bb type was used to genotype the F_2_ plants and construct a genetic map. A high-density genetic map containing 11,488 SNPs was constructed (**Figure 1**, **Table 4**, Additional file 2). The genetic map spanned 4,202.12 cM in length, ranging from 150.74 cM on A3 to 178.90 cM on A9. The SNPs were unevenly distributed on the 26 linkage groups, with only 30 SNPs on A2 and 1 318 SNPs on D5. The quality of the genetic map was analyzed by colinearity analysis, which demonstrated the accurate SNP position on the constructed genetic map (**Table 4**, **Figure 2**).

**Table 3:** Parent marker types and the number of SNPs.

Marker type	SNP number
aa×bb	410726
ab×cc	159
cc×ab	47
ef×eg	340
hk×hk	325126
lm×ll	162538
nn×np	406706

**Table 4:** Detailed information on the genetic map.

Chr.	No. of Marker	Length (cM)	Average interval (cM)	Largest gap (cM)	Coe. of collinearity
A1	165	157.86	0.96	10.09	-1.00
A2	30	161.10	5.56	28.19	-1.00
A3	487	150.74	0.31	9.25	-1.00
A4	514	164.16	0.32	10.77	-0.99
A5	488	168.17	0.35	3.43	-1.00
A6	152	162.13	1.07	14.63	-1.00
A7	583	162.98	0.28	9.13	-0.90
A8	414	156.02	0.38	7.74	-0.84
A9	624	178.90	0.29	7.61	-1.00
A10	415	161.14	0.39	8.91	-0.99
A11	338	161.48	0.48	6.49	-1.00
A12	270	160.78	0.60	13.61	-1.00
A13	1115	172.27	0.15	2.51	-0.82
At	5595	2117.74	0.74	28.19	–
D1	752	159.88	0.21	6.18	-0.68
D2	311	161.47	0.52	8.92	-1.00
D3	83	166.45	2.03	21.48	-1.00
D4	82	161.89	2.00	19.21	-1.00
D5	1318	162.01	0.12	2.60	-1.00
D6	241	170.41	0.71	13.22	-1.00
D7	359	157.39	0.44	25.66	-1.00
D8	900	155.69	0.17	2.66	-0.97
D9	349	160.44	0.46	8.78	-1.00
D10	743	161.22	0.22	9.77	-0.98
D11	236	151.28	0.64	7.66	-1.00
D12	323	161.79	0.50	17.07	-1.00
D13	196	154.46	0.79	7.64	-1.00
Dt	5893	2084.38	0.61	25.67	–
total	11488	4202.12	0.37	28.19	–

Chr., chromosome; No., number; Coe., coefficient; cM, centi morgan.

### QTL mapping

A total of 13 QTL were mapped and distributed on 11 chromosomes (**Table 5**). Two stable QTL were mapped, including *qNFFB-D3-1* and *qNFFB-D6-1* mapped in F_2:3_ and F_2:4_. JF914 conferred favorable alleles for 11 QTL, and JF173 conferred for the other two QTL. Only *qNFFB-D3-1* contributed more than 10% of the phenotypic variation. Thus, this QTL might be vital loci regulating NFFB in the tested population.

**Table 5:** Detailed information of the mapped QTL.

QTL Name	Pop	Pos (cM)	Left marker	Right marker	LOD	PV (%)	Add	Dom
*qNFFB-A4-1*	F_2_	40.00	chr4_75497483	chr4_75488413	2.81	5.03	-0.04	0.39
*qNFFB-A7-1*	F_2:4_	24.00	chr7_96247777	chr7_92676059	5.13	9.03	-0.14	0.09
*qNFFB-A7-2*	F_2:3_	28.00	chr7_92674198	chr7_92670233	3.50	6.93	-0.18	-0.01
*qNFFB-A11-1*	F_2_	3.00	chr11_119686364	chr11_119649722	4.03	8.63	-0.05	0.84
*qNFFB-D2-1*	F_2:3_	71.00	chr15_61609865	chr15_61609728	2.73	5.30	-0.13	-0.07
*qNFFB-D3-1*	F_2:3_	51.00	chr16_41836768	chr16_17130088	4.16	8.21	-0.18	0.01
	F_2:4_	50.00	chr16_41839226	chr16_41836768	5.62	10.11	-0.17	0.13
*qNFFB-D5-1*	F_2:3_	9.00	chr18_61260861	chr18_61249526	3.30	6.35	0.00	-0.47
*qNFFB-D6-1*	F_2:3_	106.00	chr19_12617417	chr19_12617401	3.34	6.52	0.06	-0.24
	F_2:4_	106.00	chr19_12617417	chr19_12617401	3.43	5.75	0.06	-0.18
*qNFFB-D7-1*	F_2:4_	95.00	chr20_15136585	chr20_14955163	4.35	7.49	-0.16	-0.02
*qNFFB-D8-1*	F_2_	139.00	chr21_5256024	chr21_5235564	3.01	5.87	-0.27	0.00
*qNFFB-D10-1*	F_2_	161.00	chr23_67766849	chr23_67763158	3.51	6.71	-0.31	-0.20
*qNFFB-D12-1*	F_2_	0.00	chr25_62606647	chr25_62552111	2.68	5.06	-0.04	-0.45
*qNFFB-D12-2*	F_2_	138.00	chr25_2807174	chr25_2714956	2.57	4.74	-0.17	-0.26

Pop, population; Pos, position; PV, phenotypic variation; Add, additive effect; Dom, dominance effect; Note: PH, plant height; NFFB, the node of the first fruiting branch; FBP, flowering-to-boll opening period; FT, flowering timing; WGP, whole growth period.

**Figure 3:**
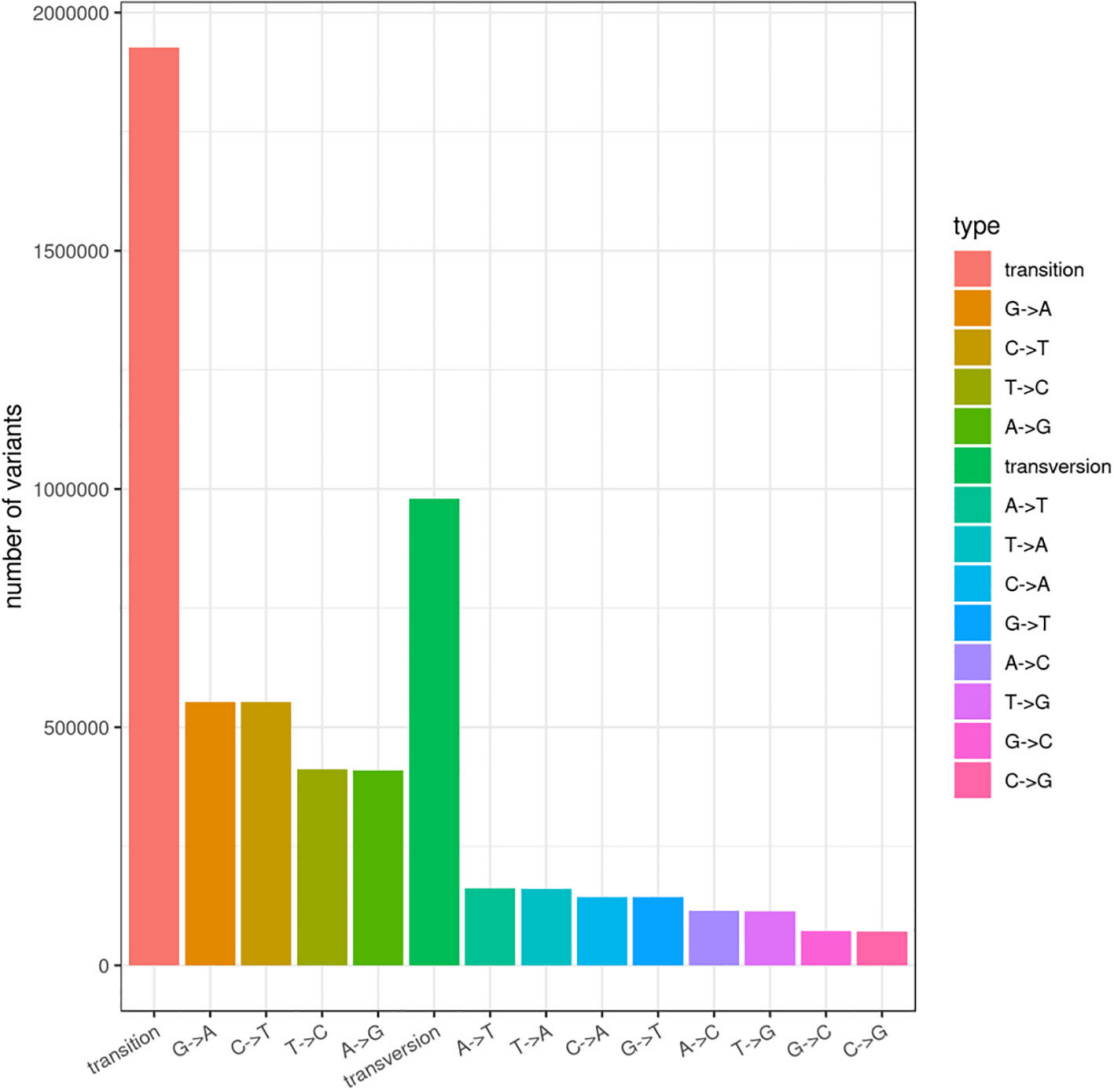
Statistic number of each SNP type.

A total of 2,907,790 SNPs were detected by BSA-seq, including 1,926,811 transition types and 979,643 transversion types (**Figure 3**). After filtration, 348,074 high-quality SNPs were reserved (Additional file 3). And SNP index was calculated for 337,651 SNPs (Additional file 4). A total of 197 and 99 regions were found through Δ(SNP-index) analysis and ED analysis, respectively (Additional file 5). Thirty-nine regions containing 2310 SNPs on 12 chromosomes were common between the results of Δ(SNP-index) analysis and ED analysis, which were recognized as the candidate regions for NFFB (Additional file 6). Two regions on D3 (41,779,195–41,836,120 bp and 41,836,768–41,872,287 bp) overlapped with the stable QTL *qNFFB-D3-1* (17,130,008–41,839,226 bp). Thus, the 24.7 Mb interval of *qNFFB-D3-1* might be shortened to the 92.4 Kb key interval.

### Candidate gene analysis

**Figure 4:**
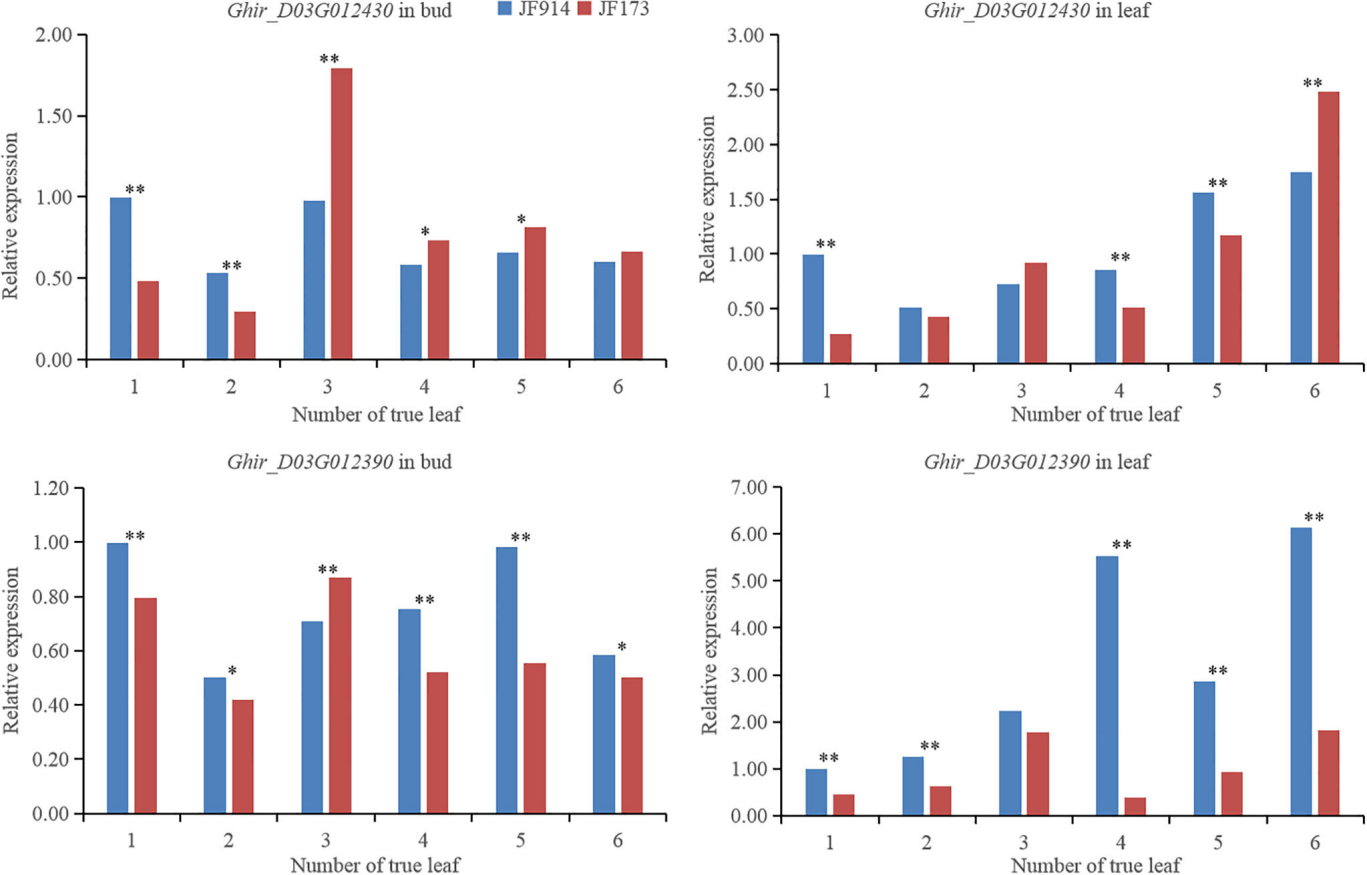
Gene expression level in the bud and leaf of JF914 and JF173. ^*^, the difference reached *p*=0.05 significance level; ^**^, the difference reached *p*=0.01 significance level.

Eight genes were annotated in the 92.4 Kb interval of the stable QTL *qNFFB-D3-1* (D3, 41,779,195–41,836,120 bp and 41,836,768–41,872,287 bp) (**Table 6**). By qRT-PCR, 2 genes showed significant different and regular expression patterns between the buds of JF914 and JF173 (**Figure 4**). *Ghir_D03G012430* expressed at a lower level at 1- and 2-leaf stages and increased sharply to a higher expression level at 3- to 6-leaf stages in the bud of JF173 than that of JF914. *Ghir_D03G012390* reached the highest expression level in the buds of JF173 and JF914 at 3- and 5-true leaf stages, respectively. The expression levels of the above mentioned genes in leaves showed no regular patterns. Thus, these 2 genes might be involved in NFFB regulation.

**Table 6:** The eight annotated genes in the 92.4 Kb interval.

Gene ID	Gene Name	Description
*Ghir_D03G012380*	Bicc1	Protein bicaudal C homolog 1
*Ghir_D03G012390*	FAM214B	Protein FAM214B
*Ghir_D03G012400*	At1g54610	Probable serine/threonine-protein kinase At1g54610
*Ghir_D03G012410*	AGAP005782	ATPase ASNA1 homolog
*Ghir_D03G012420*	SAE1B-2	SUMO-activating enzyme submit 1B-2
*Ghir_D03G012430*	pan1	Actin cytoskeleton-regulatory complex protein pan1
*Ghir_D03G012440*	HSD1	11-beta-hydroxysteroid dehydrogenase 1B
*Ghir_D03G012450*	RPL7A-2	60S ribosomal protein L7a-2

## Discussion

As a labor-intensive crop, cotton is increasingly unsuitable for manual planting in China, which raises the very pressing need for whole-process mechanization (Ma et al., 2019). Earliness is a vital trait for the practice of mechanism. Xinjiang is one of the most important cotton-growing regions in the world, accounting for 84.94% of China and ~19% of the world of cotton production (Han et al., 2020). Unstable weather conditions during the cotton planting season may cause heavy losses, especially in northern Xinjiang. Late sowing by planting early-maturity cotton is a useful method to avoid adverse weather in spring (Cheng et al., 2021). Besides, early-maturity cotton can optimize farmland cropping systems by directly planting cotton after wheat harvesting (Li et al., 2020). Thus, to improve efficiency and breed early maturity varieties suitable for mechanical harvesting, there is more need for the genetic detection of cotton earliness. In this study, an F_2_ population containing 417 plants was constructed to map QTL for cotton earliness. High-quality and density SNP markers were detected by high-throughput genome sequencing. A high-density genetic map containing 11,488 SNP and spanning 4,202.12 cM was constructed using 200 F_2_ plants, which is comparable with the genetic maps used for cotton earliness-related QTL mapping previously reported by Jia et al. (2016) (6434 loci, 4071.98 cM, 137 RILs) and Li et al. (2017) (3978 SNP, 2480 cM, 170 F_2_ plants).

The genetic basis of earliness-related traits is complex, involving WGP, FT, FBP, PH, NFFB, and HNFFB, all of which are quantitative traits controlled by large amounts of minor effect genes (Lacape et al., 2013; Su et al., 2016; Li et al., 2021). The 247 QTL reported by Jia et al. (2016) could explain 0.28–29.37% of the phenotypic variation, and 52 QTL could be detected in at least 2 years. The 47 QTL reported by Li et al. (2017) could explain 3.07–32.57% of the phenotypic variation, and none could be detected repeatedly. The SNPs for earliness traits detected by GWAS could explain 5.36%-15.56% of the phenotypic variation (Su et al., 2016). This study mapped 13 QTL with a 4.74–10.11% phenotypic variation explanation rate for NFFB. Two QTL could be detected in 2 generations, including *qNFFB-D3-1* and *qNFFB-D6-1*, and *qNFFB-D3-1* explained more than 10% of the phenotypic variation. At the same time, it is difficult to dissect the genetic basis under cotton earliness clearly, of the lack of both major and stable QTL (Li et al., 2020).

NFFB is an important index for earliness, such as in cotton (Jia et al., 2016) and pepper (Zhang et al., 2019). And NFFB was considered the most reliable and practical measurement of cotton earliness (Ray and Richmond, 1966; Guo et al., 2008). Previously, at least 80 QTL for NFFB were mapped on almost all 26 cotton chromosomes and most of these QTL have tiny genetic effect (Guo et al., 2008; Guo et al., 2009; Li et al., 2012; Jia et al., 2016; Li et al., 2017). As a typical quantitative trait, map a stable major QTL for NFFB is very precious for excavating candidate genes. The chromosome D3 was repeatedly mapped with outstanding QTL: by Jia et al. (2016); Su et al. (2016); Li et al. (2017), and Ma et al. (2018). Thus, it is interesting and hopeful that D3 contains vital genes regulating NFFB. In this study, one stable QTL *qNFFB-D3-1* was mapped in F_2:3_ and F_2:4_ generations and explained 8.21–10.11% of phenotypic variation. The confidence interval of *qNFFB-D3-1* locates between 17.1 to 41.8 Mb, spans a long region of about 24.7 Mb. QTL at this region have been reported repeatedly such as by Jia et al. (2016) (*qNFFB-D3-1*, *qNFFB-D3-2*, *qNFFB-D3-3*, *qNFFB-D3-4*), Li et al. (2017) (*qNFFB-D3-1*), Li et al. (2021) (*rsD03_39122594*), and Zhang et al. (2021) (*qNFFB-Dt3-3*). Candidate genes for cotton earliness in this region were found, such as *GhEMF2* by Jia et al. (2016) and Ma et al. (2020), *Gh_D03G0885* and *Gh_D03G0922* by Li et al. (2017), *Ghir_D03G011310* by Li et al. (2021), and *GhAPL* and *GhHAD5* by Zhang et al. (2021). Other candidate genes for earliness on chromosome D3 were reported, such as *GhCIP1* and *GhUCE* by Ma et al. (2018) and *CotAD_01947* by Su et al. (2016). Thus, it seems likely that *qNFFB-D3-1* contains candidate genes for cotton earliness.

In recent years, BSA-seq has become an efficient method in QTL mapping and functional gene mining and has been widely applied, such as in rice (Takagi et al., 2013; Zhang et al., 2021), tomato (Illa-Berenguer et al., 2015), melon (Hu et al., 2022), *Brassica napus* (Ye et al., 2022), maize (Chen et al., 2021), and cucumber (Lu et al., 2014). In cotton, genes controlling oil content (Liu et al., 2020), virescent (Zhu et al., 2017; Gao et al., 2021), nulliplex-branch (Chen et al., 2015; Wen et al., 2021), and NFFB (Zhang et al., 2021) were mapped by BSA-seq. By combining QTL mapping and BSA-seq, QTL can be finely mapped to a very small interval, significantly improving the mining efficiency of vital genes under important quantitative traits (Chen et al., 2022; Hu et al., 2022). In this study, aiming to map candidate genes for NFFB, one line from the F_2:4_ population with low NFFB and similar phenotype to JF914 was used as the maternal parent and backcrossed with JF914. A BC_1_F_2_ population containing 561 plants was constructed. A total of 60 plants with extremely high (30 plants) or low (30 plants) NFFB from the BC_1_F_2_ population were selected to construct the high and low pools. And 39 candidate regions were found by Δ(SNP-index) and ED methods. Two regions on D3 (41,779,195–41,836,120 bp, 41,836,768–41,872,287 bp) overlapped with the stable QTL *qNFFB-D3-1* (17,130,008–41,839,226 bp). Thus, the stable QTL *qNFFB-D3-1* spanning 24.7 Mb was shortened to 92.4 Kb key interval, and eight genes were annotated.

By qRT-PCR, *Ghir_D03G012430* was expressed at a lower level at 1- and 2-leaf stages and increased sharply to a higher level at 3- to 6-leaf stages in the bud of JF173 than that of JF914. *Ghir_D03G012390* reached the highest expression level in the buds of JF173 and JF914 at 3- and 5-true leaf stages, respectively. *Ghir_D03G012430* is a *pan1* gene. As reported, *pan1* functions in cell asymmetric division and development (Best et al., 2021; Lu et al., 2022). *Ghir_D03G012390* codes a FAM214B protein, which is vital in cell aging (Hernandez-Segura et al., 2017; Macedo et al., 2018). As JF173 has lower NFFB and better early maturity, the different expression patterns of *Ghir_D03G012430* and *Ghir_D03G012390* imply that they may be involved in NFFB formation and earliness regulation in cotton.

## Data availability statement

The data presented in the study are deposited in the SRA repository, accession number PRJNA821354.

## Author contributions

ML and XJ: conceived the project and set the scientific objectives. JZ, HZ, GW, and SW contributed to equipment preparation and data acquisition. XJ: wrote the manuscript. ML and GW: reviewed and edited the manuscript. All authors contributed to the article and approved the submitted version.
